# Comparing narcotics detection canine accuracy across breeds

**DOI:** 10.1016/j.heliyon.2023.e19040

**Published:** 2023-08-09

**Authors:** Brian Lee Rice, Joseph Velasco

**Affiliations:** a University of Arkansas Fort Smith, USA; b Sul Ross State University, USA

**Keywords:** Police canines, Detection canines, Canine accuracy, Detection canine breeds

## Abstract

Investigations of detection canine performance across breeds has yielded mixed results, often reporting behavioral differences observed by researchers or reported by handlers through surveys. The present study tested 34 narcotics detection canines; 25 Belgian Malinois and 9 German Shepherds to determine if there were any differences between the breeds in overall rate of detection accuracy and the number of false alerts in searches. As previously determined, results of the present study support the continued use of narcotic detection canines for law enforcement agencies (i.e., positive alert percentage >90%; false alert percentage <10%). The results indicate that Belgian Malinois and German Shepherds are statistically undifferentiated in their overall rate of narcotics detection accuracy [V(34) = 0.048, p = 0.778] and [χ^2^(1,34) = 0.080, p = 0.778]. The results further indicate that Belgian Malinois and German Shepherds are statistically undifferentiated in their number of false alerts in narcotics searches [V(34) = 0.133, p = 0.437] and [χ ^2^(1,34) = 0.604, p = 0.43].

## 1 Introduction

The olfactory abilities of canines to detect various objects, organic compounds, and organisms is well established [1,2,3]. Whereas humans have around five million olfactory receptor neurons (ORNs), canines have around 200 million [4]. Though canines have a clear olfactory advantage over humans, several studies [1,5,[2], [3], [6], [7], [8]] recognize the need to identify performance differences and working potential in canines and assess their suitability to accomplish various types of detection tasks (e.g., narcotics, cadavers, conservation/environmental). Investigations of detection canine performance and predicted suitability often address the needs of two complimentary interests: 1) the academic interest of investigating animal physiology and cognition and 2) the professional interests of selecting, preparing, or deploying canines for scent detection tasks. Of the numerous detection tasks afforded to canines, detecting narcotics is the most common [9]. Though several studies attempt to assess the performance of narcotics detection canines, few [2] investigate breed comparisons. The present study investigates narcotics detection canine accuracy across breeds to contribute to the academic literature on canine olfaction and to inform the selection of canines for narcotics detection tasks.

Though behavioral differences across breeds are often overgeneralized [10], some studies [11,12] report that breeding priorities have generated differentiation in abilities, physiology, and behavior. However, this research is tempered by studies [13] reporting significant differences in behavior within breeds. For example, a UK study [14] surveyed 244 detection canine handlers (handling 275 detection canines representing 13 different breeds) to identify the ideal breed of canine best suited for specific search tasks. Further, the handlers were asked to rate 30 behavioral and physical characteristics for their relative importance as attributes influencing the selection of detection canines across proactive drug, passive drug, and explosives detection tasks. For proactive narcotics searches (i.e., searching indoor and outdoor spaces), the English Springer Spaniel was rated as the preferred breed. For passive narcotics searches (i.e., searching people and luggage often from a leash), the Labrador Retriever was the preferred breed.

Canine olfactory performance has been linked to physiology, as head conformation (i.e., skull structure) affects the efficiency of the olfactory apparatus [15]. Though the exact measurement thresholds are not clearly defined in the literature, researchers calculate head conformation as a ratio between skull width and skull length, yielding three types of skull conformations: brachycephalic (average value = 0.81), mesocephalic (average value = 0.52), and dolichocephalic (average value = 0.39) [15]. Head conformation impacts the orientation of the olfactory bulb and the volume of the nasal cavity. Breeds with brachycephalic skulls (e.g., Boxers, English Bulldogs, French Bulldogs, Pugs, and Boston Terriers) are characterized by a compressed nasal cavity, small frontal sinuses (absent in extreme cases), and a different angle of the nasolacrimal drainage pathway as compared to different breeds [16]. While these physiological differences provide that brachycephalic skulls are positively associated with increased bite strength [17], the exaggerated body morphology of brachycephalic dogs is also associated directly or indirectly with several health disorders that affect the upper airways including brachycephalic obstructive airway syndrome [18] which may limit abilities for scent detection.

Investigations of behavioral traits associated with detection canine performance is a quickly growing body of literature. Much of the literature on canine detection performance relating to behavioral traits is established through a priori research which surveys narcotics detection handlers and or trainers. One such study [19] surveyed Transportation Security Administration (TSA) handlers for canine traits they felt were important for work with detecting narcotics and explosives. The top seven nominated traits were playfulness, human-perceived relationship as deemed by handler, human focus, calmness, concentration, activity/excitability, and search drive. In a related British study, thirty behavioral attributes were assessed across canine sex and breed [14] using a survey of 244 dog handlers and trainers of 13 different breeds. In that study, five characteristics offered significant variation (i.e., tendency to be distracted when searching, agility, motivation to obtain food, independence, and stamina). For the sample, the preferred breed was the English Springer Spaniel, followed by the Labrador Retriever.

Based on Rooney and Brashaw's [14] 30 traits, Adamkiewicz et al. [20] investigated Labrador Retrievers and German Shepherds for 28 behavioral traits associated with narcotics and explosive detection performance. Handlers and trainers rated the four most important traits across both detection specialties as (1) willingness to sniff objects, (2) concentration (focusing), (3) acuity of smell and (4) willingness to bring an object back to a person. Across the top four traits, Labrador Retrievers were rated by handlers as more ideal for narcotics detection work than German Shepherds on concentration (focusing) ability while German Shepherds received more ideal ratings from trainers than Labrador Retrievers on acuity of sense of smell. Taking all 28 traits together, both breeds were undifferentiated on trainer and handler satisfaction rate for both narcotics and explosives detection.

In the United States, Labrador Retrievers, German Shepherds, Terriers (e.g., Fox, Welsh, and Jack Russell) and English Springer Spaniels are commonly selected as breeds for narcotics detection [2]. However, the Belgian Malinois has become increasingly popular for narcotics detection tasks [21], making German Shepherds and Belgian Malinois two of the most popular narcotics detection breeds [22]. Given that narcotic detection performance is recognized to be a combination of several factors including (but not limited to) anatomical differences and behavioral traits, investigations of breed differences on narcotics detection accuracy is warranted. Therefore, the present study has two goals: (1) to examine differences in narcotics detection positive alerts across two common narcotics detection canine breeds and (2) to examine differences in narcotics detection false alerts across two common narcotics detection canine breeds. Guided by the above literature and research goals, the following hypotheses emerge.

H1 Narcotics detection accuracy is significantly related to the breed of the canine.

H2 “False alerts” in narcotics searches is significantly related to the breed of the canine.

## 2 Materials and methods

### 2.1 Canines

The present study was completed with the use of data collected from the authors’ previous research [23]. The data collection process was conducted during the time period of March to May 2020. A total of 40 canines were tested at several testing sites in Arkansas, Indiana, Louisiana, and Oklahoma. Since there was not a large enough sample for three of the breeds, this current study only includes the data from 34 canines, 25 Belgian Malinois and 9 German Shepherds (See Table 1). The lead researcher contacted training facilities in these states, and they agreed to participate in the study. Participating canines completed their initial training prior to the experiment and were either already deployed out in the field to work on the streets in a law enforcement capacity or were fully trained at a training facility but had not yet been purchased. Therefore, canines that had not yet completed their initial training were not used for this experiment. Since the tests were done with certified service canines during practices that are within the normal training activity of the canines, ethical permission was not needed. Of the 34 canines that were tested, 11 of the canines tested were from Arkansas, 11 from Indiana, 8 from Louisiana, and four from Oklahoma. Using canine teams from multiple regions helped ensure that the sample included canines and handlers from a variety of training facilities and training backgrounds.

**Table 1:** Displays the overall rate of narcotics detection accuracy by canine breed in this study.

			Overall Rate of Narcotics Detection Accuracy	
			100%	75%
Breed	MALINOIS	Count	23	2
		% within Breed	92.0%	8.0%
		% within Overall Rate of Narcotics Detection Accuracy	71.9%	100%
		% of Total	67.6%	5.9%
	GSD	Count	9	0
		% within Breed	100.0%	0.0%
		% within Overall Rate of Narcotics Detection Accuracy	28.1%	0.0%
		% of Total	26.5%	0.0%
Total		Count	32	2
		% within Breed	94.1%	5.9%
		% within Overall Rate of Narcotics Detection Accuracy	100.0%	100.0%
		% of Total	94.1%	5.9%

Though demographic information for handler and canine teams were collected, identifiable data were not collected to maintain individual and team anonymity. Information collected for each team included the state they were based in, the agency they represent (if applicable), associated training center, training classification (i.e., single-purpose vs dual-purpose), breed of canine, sex of canine, age of canine, and years of detection experience for both handler and canine. All canine teams participating in this study had a current certification by either a national canine certifying agency or by the state within which they operated. Both passive alert canines and active alert canines were allowed for this study.

### 2.2 Testing procedures

In this multi-site study, efforts were made to achieve consistency in the size of the testing rooms, types of actual narcotics, types of decoy scents, and control for residual odors. A total of eight testing sites were used in this multi-state study. There were two sites each in Arkansas, Oklahoma, Indiana, and Louisiana. The testing was conducted at training facilities that had not been used for narcotics detection training within the last seven days so that residual odors would not be present. This information was verified by the canine trainer at each location prior to testing. These safeguards should have prevented issues with false alerts related to residual odors in the testing area. Although residual odor from narcotics in the testing area would not be present after 7 days, teams were allowed to search the locations without sterilization after previous canines searched the same area that day during the testing. The scent cues left behind by canines that have already searched the area can alert the entering canines to previous areas where those canines alerted, both positive and false alerts.

Testing rooms were selected that were approximately 185 square meters or larger so that the four target odors would not overlap within the testing area. Each testing room had storage items such as lockers, desks, and chairs so that the target odors could be hidden where the canines would not come into direct contact with them during testing. The lead researcher was present to set up the testing facility, give the instructions to the canine teams, observe the canine teams perform their searches, and record observations.

Narcotics training samples were procured from each testing facility and were confirmed as actual street drugs through drug sample testing. Narcotics samples were tested by their respective state crime labs, while other samples were supplied to the testing facilities from the Drug Enforcement Agency (DEA), which ensured that all narcotics used in the testing had been verified as containing the actual target odor. Across all testing sites, marijuana, cocaine, methamphetamine, and heroin were hidden for the testing. There was one of each drug type hidden in the room, for a total of four hides in each search. The researcher labeled the marijuana as Odor 1, cocaine as Odor 2, methamphetamine as Odor 3, and heroin as Odor 4 for all testing for ease of data collection throughout the process. With each testing locating providing narcotics samples, canines were likely to be tested with familiar target ordors.

Three common decoy scents were used and placed for each testing scenario. Decoy items were dog food, plastic baggies, and dryer sheets. All three decoy scent types were used at all testing locations. New samples of each were used at each site to maintain strength of odor.

The lead researcher was the only individual who handled the decoy scents and the target odors. Handlers were not allowed to view the researcher placing the target odors in the search area. Decoy scents were placed in the room first prior to touching any target odors. Efforts were made to avoid cross-contamination of target odors when placing the items in the area by wearing nitrile gloves when touching the items and not placing the different target odors in the same location. New nitrile gloves were used for each sample as it was placed. The drug samples were in a plastic bag that remained opened during the search. The drug amounts used in this study were between 10 g and 20 g. This conforms to SWGDOG-2-SC8 (2009) guidelines that narcotics detection canine certification testing be conducted with at least 5 g of the actual substance being detected. After the drugs were placed in hidden locations, the odor was allowed to sit for at least 15 min prior to the beginning of searches so that the odor could build. Each canine and handler team was only allowed one opportunity to conduct a search in the testing area. Allowing canines to search twice could affect the results since they may remember their previous alert locations and alert based on memory alone.

The experiment was set up in a single-blind fashion. The lead researcher was the only individual who was aware of the locations of the hidden drugs. In some situations, there was a trainer on site, but they were not involved with the testing process.

Communication with the handlers was limited to obtaining consent and providing instructions prior to the beginning of the testing and verifying that they understood the testing process. The participants were given an informed consent form prior to participating in the study. Written consent was obtained from agencies involved and from the training facilities prior to testing. Each organization supplied a signed letter of cooperation that will be maintained by the lead researcher.

Improper and inconsistent handling of testing substances were mitigated by the lead researcher preparing all testing rooms across all testing locations. Similar efforts were made to provide consistency in the non-disclosure of target placements. Locations of the hidden drugs were not disclosed to participants until all teams at that location had completed the experiment. This was to provide that successive tests were not contaminated by handlers communicating with each other. The handlers remained in another location where they were unable to observe any testing area activity prior to the testing. Handlers were given instructions to call out the alerts as they occurred during the search so the researcher could document the results. Instructions were given to each canine team and each team was given the opportunity to ask any questions for clarification purposes. Once the handler acknowledged that they understood the instructions, the researcher told them to begin the search when ready. The handlers were not given confirmation when they called out each alert. They continued with their search and notified the researcher of any other alerts until they felt the search was complete. The handler was instructed to call out each alert as they believed the canine was indicating the presence of a target odor or it would not be counted. To make sure possible target odor locations was not shared, upon finishing their search, handlers were separated from the other handlers until all participants had completed their testing. Once all handlers had completed their searches, all participants were advised of the locations where target odors were placed and the weights so that they could properly document their search on a training log.

### 2.3 Statistical methods

The accuracy of each canine breed was measured by detection rate and total number of false alerts. Both factors are equally critical when determining overall accuracy of detection canines. The detection rate for this study was defined as the total number of target odors found divided by the total number of target odors hidden (4) by the canine. The false alert total is the number of false alerts made by each individual canine during the respective searches divided by the total number of target odors hidden (4). The research questions were explored using a Chi-square test of independence with calculations for Lambda and Cramer's V test to determine the measure of association between variables.

### 2.4 Ethical note

Ethical permission was not needed as the tests were done with certified service canines during practices that are within the normal training activity of the canines.

Of the three canines that detected less than 100% of narcotics, two were Belgian Malinois breeds, both three years old, with one male and one female. The female Malinois has been in service less than one year with a handler with one year of experience. It is single purpose at a training facility. The male Malinois is dual purpose at an agency. The male Malinois has been in service for one year and the handler has one year of experience. A five year old male was the lone German Shepherd to detect less than 100%.

Two canines had false alerts, one Belgian Malinois and one German Shepherd. The Belgian Malinois was a dual purpose, three-year old male from an agency with one-year experience and whose handler had the same experience. The German Shepherd was a dual purpose, four-year old male from an agency with two-years of experience and whose handler had the same experience level.

A Pearson chi-square test of independence with Cramer's V was used to test the first hypothesis related to breed of canine and detection accuracy. The null hypothesis was accepted [V(34) = 0.048, p = 0.778] and [χ^2^(1,34) = 0.080, p = 0.778] with 95% CI (0.055, 8.748) = 0.696. Therefore, Belgian Malinois are 69.6% more likely to alert at 100% than German Shepherds. There was no statistically significant relationship between the canine breed and the overall rate of narcotics detection accuracy (see Table 2). Directional measures for the proportional reduction in error further indicate that narcotics detection accuracy is not associated with canine breed among Belgian Malinois and German Shepherds (lambda = .000) with a p-value not able to be computed because the asymptotic standard error equals zero (see Table 3).

**Table 2:** Measures of association: Canine breed * overall rate of narcotics detection accuracy.

Measure of Association	Value	N	df	Asymptotic Significance (2-sided)	Approximate Significance
Pearson Chi-Square	.080^a^	34	1	0.778	–
Lambda	0.000	–	–	–	b
Cramer's V	0.048	34	–	–	0.778

a 8 cells (80.0%) have expected count less than 5. The minimum expected count is 0.08.

b Cannot be computed because the asymptotic standard error equals zero.

**Table 3:** Indicates the overall number of false alerts by canine breed in this study.

			Number of False Alerts in Narcotics Searches	
			0	1
Breed	MALINOIS	Count	24	1
		% within Breed	96.0%	4.0%
		% within Number of False Alerts in Narcotics Searches	75.0%	50.0%
		% of Total	70.6%	2.9%
	GSD	Count	8	1
		% within Breed	88.9%	11.1%
		% within Number of False Alerts in Narcotics Searches	25.0%	50.0%
		% of Total	23.5%	2.9%
Total		Count	32	2
		% within Breed	94.1%	5.9%
		% within Number of False Alerts in Narcotics Searches	100.0%	100.0%
		% of Total	94.1%	5.9%

A Pearson chi-square test of independence with Cramer's V was used to test the second hypothesis regarding canine breed and false alerts. The null hypotheses was accepted [V(34) = 0.133, p = 0.437] and [χ^2^(1,34) = 0.604, p = 0.437], demonstrating that Belgian Malinois and German Shepherds were statistically undifferentiated on false alerts of narcotics (See Table 4). With a 95% CI (0.168, 53.71) = 3.0, Belgian Malinois are at 3 times the risk of False Alerts than German Shepherds. Directional measures for the proportional reduction in error further indicate that narcotics detection false alerts is not associated with canine breed amongst Belgian Malinois and German Shepherds (lambda = 0.000) with a p-value not able to be computed because the asymptotic standard error equals zero.

**Table 4:** Measures of association: Canine breed * number of false alerts in narcotics searches.

Measure of Association	Value	N	df	Asymptotic Significance (2-sided)	Approximate Significance
Pearson Chi-Square	0.604^a^	34	1	0.43	–
Lambda	0.105	–	–	–	0.525
Cramer's V	0.133	34	–	–	0.437

a 2 cells (50.0%) have expected count less than 5. The minimum expected count is 0.53.

## 3 Discussion

The suitability of particular breeds for narcotics detection was also researched previously by Jezierski et al. [2]. In that study, German Shepherds proved to be the superior breed with a detection rate of approximately 87% and a false alert rate of 8%. The researchers did state that many times canines are selected due to breed preference, availability, and current opinions. In this current study, German Shepherds had an accuracy rate of 100%, but it must be mentioned that only 9 of the 34 canines being tested were of this breed. Two of the Belgian malinois missed one target odor each, but they were the largest sample tested with a total of 25 canine teams. The accuracy rate of the Belgian malinois was approximately 98%. Also, the German Shepherds in this study had a false alert rate of 11%, which is very close to the 8% rate found by Jezierski et al. [2]. The Belgian malinois had a false alert rate of only 4Ultimately, this current study revealed no major differences in accuracy rates or false alert rates between the breeds. This is important for law enforcement agencies to know that a focus on individual breeds is not necessary when they are in the process of purchasing canines from facilities.

The present study is not without limitations. The use of sterile, non-operational environments to conduct assessments may be problematic in that multiple variables present in the field may exert their influence in a way that a sterile laboratory setting may not capture [24]. However, it should be taken into consideration that certifications of narcotics detection canines are not conducted in a laboratory setting. A second but related limitation is that the canine/handler teams were allowed to search testing locations one after another without subsequent sterilization. There is the potential that canines leave behind saliva at alert locations or locations where they stop during the search, possibly leaving cues for other dogs during their searches. Third, the sample of canines was not balanced by sex and breed. A larger sample may have produced different results.

Future research could include a larger sample of canines across breeds that would focus on accuracy and false alert rates of rescue canines that have transitioned into narcotics detection work, as their use in the field is becoming more common. Future research could investigate performance differences across canines selected for detection work at an early age versus canines repurposed at a later life stage Longitudinal studies of canine detection performance could be warranted to investigate changes in accuracy and false alert rates over time as canine/handler teams gain more experience in training and in the field. Investigations of interaction effects of sex, age, and breed are also warranted. Lastly, olfactory performance in narcotics detection across skull morphology is needed to address real-world performance concerns for practitioners and to contribute to the literature on canine physiology and olfaction.

## 4 Conclusion

As previously determined, results of the present study support the continued use of narcotic detection canines for law enforcement agencies (i.e., positive alert percentage >90%; false alert percentage <10%). The present study suggests that there is no difference in positive alert rates among German Shepherds and Belgian Malinois breeds when the canines are tested in their normal training environments. However, the current study did determine that German Shepherds and Belgian Malinois are statistically undifferentiated in the number of false alert searches in narcotics searches. Only 4% of Belgian Malinois had false alerts compared to 11% of German Shepherds. It is notable that a dolichocephalic breed (i.e., German Shepherd) had an undifferentiated performance with a mesocephalic breed (i.e., Beligian Malinois) as skull morphology in terms of length of nasal cavity and its effects on scent detection welcome further exploration. For practitioners, the present study does not support claims that the Belgian Malinois or the German Shepherd breed are superior to the other in narcotics scent detection.

## Author contribution statement

Brian Rice; Joseph Velasco: Conceived and designed the experiments; Performed the experiments; Analyzed and interpreted the data; Contributed reagents, materials, analysis tools or data; Wrote the paper.

## Declaration of competing interest

The authors declare that they have no known competing financial interests or personal relationships that could have appeared to influence the work reported in this paper.
